# Supplementary material for: Consistency between inter-institutional panels using a three-level Angoff-standard setting in licensure tests of foreign-trained dentists in Sweden: A cohort study
Source: PLoS One. 2024 Nov 8;19(11):e0313476. doi: 10.1371/journal.pone.0313476 (PMC11548739; doi:10.1371/journal.pone.0313476)
Supplement: Attachment — Submitted filename: Response to reviewers.docx [file pone.0313476.s002.docx]

PONE-D-24-15178

Consistency between inter-institutional panels using a three-level Angoff - standard setting in licensure tests of foreign-trained dentists in Sweden

**Reviewer #1:**

Abstract

- Please revise, and stick to Journal style.

- No subheadings mandatory. Please consult some recently published Plos One papers.

<https://journals.plos.org/plosone/article?id=10.1371/journal.pone.0306863>

https://journals.plos.org/plosone/article?id=10.1371/journal.pone.0306863

- Please provide exact P values here. Remember that terms like "very strong relationship" and "scores were overall lower" would seem too vague, and do not provide sound information.

- With your revision, please stick to the allowed word maximum, to provide as much information as possible.

Comment: We have revised the abstract to conform with the journal's style. We have used the recently published article in formatting our manuscript. The exact p-values are now provided in the abstract.

Intro

- "To address this, the present study aims to investigate (...)." This study has been finished hasn't it? Please switch to past tense.

- No subheadings with this section.

- Please elaborate bot aims and objectives with this section. Do not provide a literature review here.

- A sound and valid null hypothesis is missing at the end of this section.

Comment: The Introduction has been revised to reflect that the study is finished. We have removed the subheadings from the Introduction to align with the journal's style requirements. The null hypothesis has been explicitly stated at the end of the Introduction. Additionally, aims and objectives have been elaborated.

Meths

- "(...) by two separate panels; The reference panel was (...)." Please revise for typos.

- Details of statistical program missing.

Comment: We have revised the text to correct any typographical errors. We have included detailed information about the statistical software used in the analyses, including the version, manufacturer, and location.

Results

- With you full text, please provide exact results. See "weakest relationship" or "moderate relationship" - you surely will agree that this would seem hard to understand.

Comment: The exact results have been provided throughout the text.

Disc

- Stick to H0 when starting this section.

- This section would seem perfectible. There would be many more aspects to discuss. Please revise carefully.

Comment: We have revised the Discussion section to focus on the null hypothesis and expanded the discussion to cover additional relevant aspects.

Concl

- With your revision, please exclusively stick to your aims.

Comment: The Conclusion has been revised to strictly adhere to the study's aims.

Refs

- This list must be adopted to Journal style.

Comment: References have been reformatted to comply with the journal's guidelines.

In total, this submission would seem interesting, is considered easily intelligible, and should be worth following after revision, depending on the latter's quality. Please stick to the comments provided above, and revise carefully.

# **Reviewer #2:**

Title page:

- It would be helpful to potential readers if the title of the publication already indicated the type of study that was involved in the publication.

- Please note the formal requirements of PLoS One regarding the font size and font type of your headings, please see "Abstract".

- Please use either American or British English in your manuscript, but do not mix the two.

Comment: Thank you for your suggestion. We have revised the title to reflect the nature of the study more clearly. We have reviewed and adjusted the font size and type of all headings throughout the manuscript to comply with PLoS One formatting guidelines. We have also revised the text to British English.

Introduction:

- "To address this, the present study aims to investigate the consistency of three-level Angoff ratings of the proficiency test between two independent panels of raters from two universities." - "To address this, the aim of the present study was to investigate..."

- Please provide a sound and valid null hypothesis at the end of your Introduction section, which should be discussed at the beginning of the Discussion section.

Comment: We have revised the text in the introduction. The null hypothesis has been explicitly stated at the end of the Introduction. Additionally, aims and objectives have been elaborated. We have revised the Discussion section to focus on the null hypothesis and expanded the discussion to cover additional relevant aspects.

Materials and methods:

- With the material used (i.e., the statistical software), please use general names with your text, followed by (brand name; manufacturer, city, ST[ate - abbreviated, if US], country) in parentheses. Stick to semicolon.

- Which test did you use to determine the (missing) normal distribution?

- The significance level α is missing. I guess it was 0.05?

- The ethics committee vote date is missing.

Comment: We have revised the Material and Methods section. We now include the brand, manufacturer, and location details for the statistical software used, as suggested. No formal test of possible normal distribution was made. Instead, we concluded that the normal distribution would be a poor model based on the way total scores were calculated. This has been clarified in the manuscript. We have revised the text to include the significance level. The date of the ethics committee approval has been included in the manuscript under Ethical consideration.

Discussion:

- The mentioning of statements regarding the null hypothesis at the beginning of this section is missing. Remember that the null hypothesis can be rejected or not rejected.

Comment: The Discussion begins with a reference to the null hypothesis. See revisions in first section of Discussion.

References:

- Please follow the author guidelines for ALL your References and provide all necessary data such as the correct abbreviated journal name, volume, issue, page numbers etc. Please carefully revise your data.

- Please make sure the title of each reference you access online is complete, correct, and includes access date and link.

- Please provide the correct and complete names of authors of websites used, e.g. References 2-4.

Comment: All references have been revised to follow the journal’s style, including online access information where relevant

# **Reviewer #3:**

The authors present valuable insight into the Swedish process of re-certifying dentists trained outside the EU. Two expert panels assessed three different types of examinations using a modified Angoff method for setting exam pass-marks. The results suggest an overall high correlation between both (expert) groups in the majority of items although the syllabus is variable from one university to another. It was concluded that simplification of the Angoff method is appropriate, less time consuming and resource-saving compared to the originally described procedure.

The paper is well organized and concise. However, information is provided in a cumbersome style, which is hard to read and confusing at times. To me the paper needs extensive linguistic revision especially in the Materials and Methods and Results sections. There is a lack of clarity, consistency and readability in particular.

• For the above mentioned reasons the abstract needs to be rewritten. The results are almost incomprehensible. Who are the participants in this study? I assume you refer to the external panel but this is nowhere mentioned and confuses the reader to the maximum.

Comment: The abstract and Methods section has been rewritten for clarity.

Why do you consider the modified Angoff method to be “promising” compared to the original one when you have not even investigated both methods?

Comment: We have clarified why the modified Angoff method is considered promising, despite not directly comparing it to the original method. See Discussion and limitations section. We have also revised the conclusions regarding the reference to the original Angoff method.

• In the Results section, the paragraph starting on page 6 l151-page 7 l158 is incomprehensible at all and probably needs visualisation. (For you who are dealing with these closely related technical terms on a daily basis this all might be self-explanatory which is obviously not the case for your readers).

Comment: We have revised the results section, adding an explanatory text in the beginning of each paragraph in the results section.

• Description of the study’s limitations is by far the best paragraph in the paper which arises the question whether an overall “similarity” (since agreement was not assessed in the first place) between to different panels assessing pass-marks of three exams is worth to be reported in a high-standard international publication?

Comment: The Discussion section has been revised to ensure the study’s contribution is clear, in the context of the study’s limitations.

• The closing sentence again refers to the traditional Angoff method which again has not been investigated in this study.

Comment: The Conclusion has been revised to remove ambiguous references to the traditional Angoff method and focuses solely on the findings.
