# Supplementary material for: Consistency between inter-institutional panels using a three-level Angoff-standard setting in licensure tests of foreign-trained dentists in Sweden: A cohort study
Source: PLoS One. 2024 Nov 8;19(11):e0313476. doi: 10.1371/journal.pone.0313476 (PMC11548739; doi:10.1371/journal.pone.0313476)
Supplement: Attachment — Submitted filename: PONE Response to reviewers 2nd revision.docx [file pone.0313476.s003.docx]

PONE-D-24-15178R1

Consistency between inter-institutional panels using a three-level Angoff - standard setting in licensure tests of foreign-trained dentists in Sweden: A cross-sectional study

**Revised title:** Consistency between inter-institutional panels using a three-level Angoff-standard setting in licensure tests of foreign-trained dentists in Sweden: A cohort study

PLOS ONE

------------------------------------------------------------------------------------------------

Dear Editor and reviewers,

Thank you for taking the time to review and to improve upon our manuscript. In the following text you will find our point-by-point response to the comments. We have also included an updated point-by-point response to the previous comments at the end of the document.

The manuscript has been formatted according to reference article: <https://journals.plos.org/plosone/article?id=10.1371/journal.pone.0228249>

------------------------------------------------------------------------------------------------

Additional Editor Comments:

- Unfortunately, the Co-Authors have not provided a point-by-point response to the previous Reviewers’ comments. Please go back to the recent recommendations, and clarify how you have responded to the recently forwarded recommendations.

**Reply:** Point-by-point response to the first review is provided in the uploaded document *PONE Response to reviewers 1st revision - point-by-point*.

- „Angoff - standard“ – please double check for correct spacebar use.

**Reply:** Thank you, this is corrected.

- „The reference panel ratings originated from the panel used to consecutively set the pass mark for regular examinations. The external panel was recruited, as part of the study, from a dental school at a university in another region in Sweden after the examinations.“ Please note that this would seem hard to understand; it would seem that you have compared regular students from two different universities. In particular when reflecting on your thoughts given with your Abstract section („This study aimed to determine the consistency of ratings by independent panels at two Swedish universities in re-certification exams for non-EU trained dentists“), this should be clarified. Please clearly define your reference and external groups.

**Reply**: The abstract has been rephrased to clarify the design of the study.

- The Co-Authors state that „A cross-sectional design was used to analyze rating consistency of two separate panels.“ Please note that a cross-sectional study design is a type of observational study design. First, this would call for pre-registration (as with all observational studies, see <https://www.ncbi.nlm.nih.gov/pmc/articles/PMC2952011/> ). Second, cross sectional studies should be reported according to STROBE (see <https://www.equator-network.org/wp-content/uploads/2015/10/STROBE_checklist_v4_cross-sectional.pdf>), along with a dedicated information given with your manuscript. You surely will agree that both aspects would seem missing with your revised and re-submitted draft.

**Reply:** Yes, we agree. We acknowledge that cross-sectional studies are observational by nature and agree that pre-registration of medical studies and adherence to STROBE guidelines are important. This study was not pre-registered. A STROBE Statement has been added at the end of ethical consideration, and a document how the manuscript adheres to the STROBE guidelines is also added to the supplementary material. (p x, line xx)

- „The panellists in the reference panel were recruited consecutively before each examination during 2019-08-01 to 2020-08-19 (...).“ With reference to the thoughts given above, this would be a group of people who share a common characteristic or experience within a defined period (e.g., have been examined. This usually would be a cohort. Consequently, this would be a cohort study comparing two subgroup cohorts (reference and external). Please double check, and give reasons why you have called this study a cross sectional study.

**Reply**: Thank you for pointing this out, we have adjusted the text accordingly in the title and in the first sentence of the methods section.

- „The two panels consisted primarily of women.“ Please provide reasons.

**Reply:** Thank you for your comment. The absolute majority (at least 75%) of the dental teachers are women in both dental schools involved. This is reflected by the sex distribution of the panels. We have added text “The two panels consisted primarily of women, which reflects the current sex-distribution of dental teachers in both universities.” In the beginning of the Results. Page 7, Row number 165-166.

- With your Conclusions, please stick exclusively to your aims (these were „This study aimed to determine the consistency of ratings by independent panels (...)“, or „This study aimed to determine whether the expectations of minimally qualified dentists are aligned, as reflected by the ratings from two universities using the three-level Angoff method“). Do not simply repeat your results here. Do not speculate. Instead, provide a reasonable and generalizable extension of your outcome.

**Reply:** Thank you, the Conclusions have been revised accordingly.

- „Additional studies are needed to fully understand the pros and the cons of the three-level Angoff method.“ This is not considered a conclusion from your study. Please, these aspects might be discussed with your Discussion section; please delete these thoughts from your conclusions.

**Reply:** Thank you for your comment, the statement has been removed from the conclusion section.

- References still have not been adapted to Journal style (please see previous comments, and compare your responses: „References have been reformatted to comply with the journal's guidelines“, and „All references have been revised to follow the journal’s style“). With your revisions, you surely want to make up this leeway. Please revise for English titles, and provide doi numbers.

**Reply:** Thank you for your comment, the references are now formatted correctly

Reviewer #1: The Co-Authors have satisfyingly revised their previous draft. Unfortunately, doi numbers still would seem missing with most of the references, but this should be perfectible in cooperation with the typesetter.

**Reply**: Thank you for your comment, the references are now formatted correctly

Reviewer #2: Dear Authors,

Abstract:
- No subheadings are required in the Abstract. Please note the comment from Reviewer #1 in the last review.
<https://journals.plos.org/plosone/article?id=10.1371/journal.pone.0310004>
<https://journals.plos.org/plosone/article?id=10.1371/journal.pone.0228249>

**Reply**: Thank you for your comment. Subheadings have been deleted in the abstract

- Please put a space before and after a mathematical operator (e.g., minus, plus, greater than, less than) in the whole manuscript as well as before and after the hyphen.

**Reply:** Thank you for your observation, this has been corrected in the text.

- Please note the comment from Reviewer #1 regarding the mention of exact p values.

**Reply:** Yes, we have corrected this by adding exact p-values in the Tables.

- Shouldn't the order of the p and Spearman's Rho values be the other way around (significance/correlation) according to your explanation? - "There was a statistically significant and strong correlation (ρ ≥ 0.70, p ≤ 0.05) between ..." - "There was a statistically significant and strong correlation (p ≤ 0.05, ρ ≥ 0.70) between..."

**Reply:** Thank you for noticing, we have corrected these sentences.

- Please report exact p values (e.g., p = .015, p < .XYZ), unless p is < .001. In accordance with the APA style, p values smaller than p < .001 are written as p < .001 (<https://www.graphpad.com/support/faq/how-to-report-p-values-in-journals>).

**Reply** Yes, we have corrected this by formatting the text and adding exact p-values in the Tables.

- "There was a statistically significant and strong correlation (ρ ≥ 0.70, p ≤ 0.05) between the ratings of the two
independent panels, with no significant differences ..." - Here, the exact p value is missing as well.

**Reply:** Thank you for observing this. In running text, we refer to overall results. The p-values have been corrected. Exact p-values have been added in the revised Tables for reference.

- Please re-check your punctuation skills in English.

**Reply:** We have re-checked the punctuation, and hope this is OK now

Introduction:
- "The null hypothesis was that there will be no significant difference..." - "The null hypothesis was that there would be no significant difference..."

**Reply** Thank you, the text has been revised according to your comment.

Materials and methods
- Please put a space before and after a mathematical operator (e.g., minus, plus, greater than, less than) in the whole manuscript as well as before and after the hyphen.

**Reply:** We have checked the manuscript and have hopefully corrected these mistakes.

- Please consistently omit the zero before the decimal point for the p values and the correlation coefficient because the statistic cannot be greater than 1 (APA style), and please do not change the notation within the manuscript.

**Reply:** We have checked the manuscript and have hopefully corrected these mistakes.

Results
- Please put a space before and after a mathematical operator (e.g., minus, plus, greater than, less than) in the whole manuscript as well as before and after the hyphen.

**Reply:** We have checked the manuscript and have hopefully corrected these mistakes

- Please report exact p values (e.g., p = .015, p < .XYZ), unless p is < .001. In accordance with the APA style, p values smaller than p < .001 are written as p < .001 (<https://www.graphpad.com/support/faq/how-to-report-p-values-in-journals>).

**Reply:** We have checked the manuscript and have hopefully corrected these mistakes

- Please consistently omit the zero before the decimal point for the p values and the correlation coefficient because the statistic cannot be greater than 1 (APA style), and please do not change the notation within the manuscript.

**Reply:** We have checked the manuscript and have hopefully corrected these mistakes

Conclusion
This chapter is formulated in very general terms.

**Reply:** The conclusion section has been rewritten in order to provide a reasonable and generalizable extension. P x line xx)

Refs
- Please revise this part with regard to up-to-dateness. For example, complete information is available on PubMed for Reference 1. <https://pubmed.ncbi.nlm.nih.gov/36039793>
I assume that it is the task of the authors to ensure that a revised version is up-to-date and free of errors.

**Reply:** We’re sorry about these mistakes, and the reference list has been updated accordingly.
